# Phrenic nerve block caused by interscalene brachial plexus block: breathing effects of different sites of injection 

**DOI:** 10.1186/s12871-016-0218-x

**Published:** 2016-07-29

**Authors:** Lars Bergmann, Stefan Martini, Miriam Kesselmeier, Wolf Armbruster, Thomas Notheisen, Michael Adamzik, Rϋdiger Eichholz

**Affiliations:** 1 Klinik fϋr Anästhesiologie, Intensivmedizin und Schmerztherapie, Knappschaftskrankenhaus Bochum Langendreer, Ruhr-Universität Bochum, In der Schornau 23-25, 44892 Bochum, Germany; 2 Clinical Epidemiology, Integrated Research and Treatment Center - Center for Sepsis Control and Care (CSCC), Jena University Hospital, Jena, Germany; 3 Klinik fϋr Anästhesiologie, Intensivmedizin und Schmerztherapie, Evangelisches Krankenhaus Unna, Unna, Germany; 4 Abteilung für Anästhesiologie, Intensivmedizin und Schmerztherapie, Berufsgenossenschaftliche Unfallklinik Tϋbingen, Tϋbingen, Germany

**Keywords:** Injections site, Interscalene block, Phrenic nerve, Breathing effects

## Abstract

**Background:**

Interscalene brachial plexus (ISB) block is often associated with phrenic nerve block and diaphragmatic paresis. The goal of our study was to test if the anterior or the posterior ultrasound guided approach of the ISB is associated with a lower incidence of phrenic nerve blocks and impaired lung function.

**Methods:**

This was a prospective, randomized and single-blinded study of 84 patients scheduled for elective shoulder surgery who fullfilled the inclusion and exclusion critereria. Patients were randomized in two groups to receive either the anterior (*n* = 42) or the posterior (*n* = 42) approach for ISB. Clinical data were recorded. In both groups patients received ISB with a total injection volume of 15 ml of ropivacaine 1 %. Spirometry was conducted at baseline (T_0_) and 30 min (T_30_) after accomplishing the block.

Changes in spirometrical variables between T_0_ and T_30_ were investigated by Wilcoxon signed-rank test for each puncture approach. The temporal difference between the posterior and the anterior puncture approach groups were again analyzed by the Wilcoxon-Mann-Whitney test.

**Results:**

The spirometric results showed a significant decrease in vital capacity, forced expiratory volume per second, and maximum nasal inspiratory breathing after the Interscalene brachial plexus block; indicating a phrenic nerve block (*p* <0.001, Wilcoxon signed-rank). A significant difference in the development of the spirometric parameters between the anterior and the posterior group could not be identified (Wilcoxon-Mann-Whitney test). Despite the changes in spirometry, no cases of dyspnea were reported.

**Conclusion:**

A different site of injection (anterior or posterior) did not show an effect in reducing the cervical block spread of the local anesthetic and the incidence of phrenic nerve blocks during during ultrasound guided Interscalene brachial plexus block. Clinical breathing effects of phrenic nerve blocks are, however, usually well compensated, and subjective dyspnea did not occur in our patients.

**Trial registration:**

German Clinical Trials Register (DRKS number 00009908, registered 26 January 2016).

## Background

Over the last 15 years, ultrasound guided peripheral nerve block techniques have grown increasingly popular in regional anesthesia in addition to landmark guided techniques and neurostimulation [1, 2]. The brachial plexus and the phrenic nerve can be depicted quite well sonographically in the interscalene region [3]. Interscalene brachial plexus blocks (ISB) only using nerve stimulation or eliciting paresthesias are associated with a high rate (up to 100 %) of phrenic nerve blocks [4–6]. Two possible reasons for this are either a C_3_, C_4_ and C_5_ nerve root block, caused by cranial spread of the blind injection of a high volume of local anesthetic, or a direct phrenic nerve block within the anterior scalene muscle fascia [7, 8]. Due to unilateral diaphragmatic paresis, the mechanics of breathing can be considerably impaired; thus, patients with impaired pulmonary function are often advised against interscalene blocks [9]. Studies have shown phrenic nerve blocks can be significantly reduced by decreased local anesthetic volumes, lower volumes usually require using ultrasound guided technique [6, 10].

The objective of this study is to investigate if the anterior ultrasound guided anterior puncture approach compared to the posterior approach of the C_5_, C_6_, C_7_ nerve roots reduces the frequency of unintentional phrenic nerve blocks measured by spirometry. The rationale for the comparison of the anterior versus posterior approach is based on the idea, that anatomically the posterior site of injection has a greater distance to the phrenic nerve than the anterior site. Additionally, using the anterior approach, the local anesthetic can easily spread along the fascia of the anterior scalene muscle and block the phrenic nerve on its course to the chest. Different rates of hemidiaphragmatic paresis could support new recommendations on how to perform ultrasound guided interscalene brachial plexus blocks.

## Methods

This prospective, single-center, randomized, single- blinded, parallel study was conducted in accordance with the ethical principles of the Helsinki Declaration, and was approved by the Ethics Committe oft he Eberhard Karls University in Tuebingen (Germany) (Research Ethics Committee No. 579/2010B01). This study was registered at the German Clinical Trial Register (DRKS number 00009908, registered 26 January 2016). URL: https://drks-neu.uniklinik-freiburg.de/drks_web/navigate.do?navigationId=trial.HTML&TRIAL_ID=DRKS00009908.

A total of 84 patients scheduled for elective shoulder surgery gave written informed consent and were included in this study. The criteria for exclusion were age <18 or >70 years, ASA status > II, an allergy to the local anesthetic used, psychiatric disorders, and pre-existing lung disease (GOLD > II) [11]. The spirometric examination and the nerve block were always performed by the same anesthesiologist. A pre-procedural ultrasound scan showing unequivocal identification of patients’ C_5_, C_6_, C_7_ nerve roots, increasing the likelihood of a well-targeted block, in each patient was also a prerequisite. All data were collected from a questionnaire which was filled out by the investigator.

### Sample size calculation and randomization

A sample size (2 × 36 patients) allows for detecting a between-group mean difference of 0.75 (standardized effect in units of standard normal distribution; a moderate to strong effect using Cohnes classification) with a comparison-wise power of 88 % at a two-sided significance level α = 5 % (planning for Student’s t-test). Addressing a potential drop-out rate, we decided to randomize an additional 2 × 7 patients (drop-out rate of ~15 %). A total sample size of 2 × 42 patients has 80 % power to detect a between-group mean difference of 0.62, which is still a relatively large (but likely clinically relevant) effect. The 84 patients were allocated via block randomization with a block size of six to either one of the groups, realized by 84 similar looking and closed envelopes.

### Anesthesiologic procedure

All patients were studied without sedation in order to avoid any interference in the spirometric tests. Venous access and patient monitoring were established (ECG, pulsoximetry and non-invasive blood pressure). ISB was performed exclusively by two staff anesthetist only, both being experts in ultrasound guided procedures.

### First spirometric examination

Following randomization a baseline spirometry measurement (Masterscope PC, Carefusion, Heidelberg, Germany) was performed on patients in the sitting position with the upper part of the body in an upright position. The lung function tests comprised the vital capacity (VC [l]), forced expiratory volume per second (FEV_1_ [l/s]), and maximum nasal inspiratory breathing maneuver (Sniff _PmaxPeak_ [kPa]). The best values (VC, FEV1 and Sniff _PmaxPeak_) were determined from three repeated measurements.

### Anterior approach

**Fig. 1:**
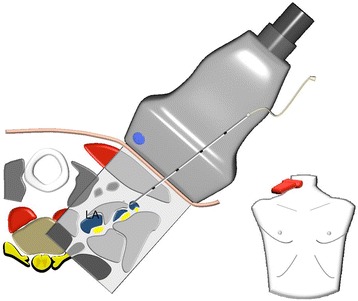
Anterior approach. *Right side*, cranial view. *Blue color* depots present the local anesthetic (LA) administered to the nerve roots C_5_, C_6_, C_7_

The skin was anesthetized under ultrasound surveillance to ensure that there was no spread of any local anesthetic below the sternocleidomastoid muscle, potentially causing unintended direct phrenic nerve block. Thereafter, we performed an ultrasound guided insertion of a cannula (Stimuplex A, 21 G, B.Braun, Melsungen, Germany) between the anterior scalene muscle and the C_5_, C_6_, C_7_ nerve roots. After having positioned the needle tip in front of the C_7_ nerve root, an injection of 5 ml ropivacaine 1 % was administered. The same needle tip position and injection volume was used for both C_6_ and C_5_, resulting in a total injection volume of 15 ml. In cases where the nerve roots were moved away from the needle tip as a result of the local anesthetic injection, corrections to the needle position were explicitly allowed ensuring direct contact of the local anesthetic to the nerve roots (Fig. 1).

### Posterior approach

**Fig. 2:**
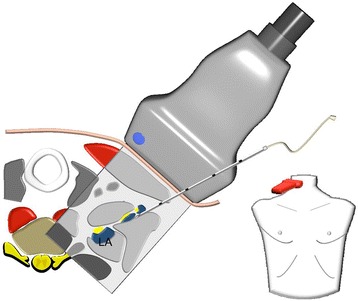
Posterior approach. *Right side*, cranial view. *Blue color* depots present the local anesthetic (LA) administered to the nerve roots C_5_, C_6_, C_7_

The skin was anesthetized under ultrasound surveillance to ensure that there was no spread of any local anesthetic below the sternocleidomastoid muscle, potentially causing unintended direct phrenic nerve block. Thereafter, we performed an ultrasound guided insertion of a cannula (Stimuplex A, 21 G, B.Braun, Melsungen, Germany) between the median scalene muscle and the C_5_, C_6_, C_7_ nerve roots. After having positioned the needle tip posteriorly of the C_7_ nerve root, an injection of 5 ml ropivacaine 1 % was administered. The same needle tip position and injection volume was used for both C_6_ and C_5_, resulting in a total injection volume of 15 ml. In cases where the nerve roots were moved away from the needle tip as a result of the local anesthetic injection, corrections to the needle position were explicitly allowed ensuring direct contact of the local anesthetic to the nerve roots (Fig. 2).

After the ISB was performed, using either approach, a subcutaneous infiltration of 5 ml ropivacaine 1 % administered along the clavicle up to the acromion was done to block the suprascapular nerves in addition to the interscalene brachial plexus.

### Follow-up spirometric examination

The second spirometric examination of the patients was carried out 30 min after implementation of the interscalene block. Patient posture and spirometric tests were identical to the first spirometric examination.

A decrease of the VC, FEV_1_ or the Sniff _PmaxPeak_ by >20 % following the first baseline spirometry was defined as a criterion for a hemidiaphragmatic paresis [11].

### Sedation

After the follow-up spirometry was completed, all the patients received 0.033 mg/kg body weight midazolam, 0.067 μg/kg body weight sufentanil, and 0.0133 mg/kg body weight propofol for sedation. To lower blood pressure, clonidine was administered when necessary up to a maximum dose of 300 μg intravenously. All operations were planned without general anesthesia. Subsequently, the unplanned need for general anesthesia was used as a criterion to evaluate the block success rate.

### Statistics

Patients’ characteristics were summarized by quartiles in case of quantitative continuous variables or by relative and absolute frequencies in case of count variables. To verify the randomization, we performed explorative comparisons between groups (i.e. the two differing approaches) using Wilcoxon-Mann-Whitney tests for quantitative continuous variables or Fisher’s exact tests for count variables; we observed no evidence for baseline differences.

Changes in spirometrical variables between baseline (T_0_) and 30 min later (T_30_) were analyzed by Wilcoxon signed-rank tests for each puncture approach. The temporal difference (D = T_0_-T_30_) between the posterior and the anterior puncture approach groups (D_posterior_-D_anterior_) were again investigated by Wilcoxon-Mann-Whitney tests. As sensitivity analysis, we additionally compared the posterior and the anterior puncture approach groups at both time points (T_0_ and T_30_) by Wilcoxon-Mann-Whitney tests. We applied a two-sided significance level α = 5 %, did not correct for multiple testing and report two-sided p-values. 95 % confidence intervals (95 % CI) were derived with normal approximation. 95 % CI for binomial proportions are Clopper-Pearson-Intervals. All analyses were performed using R version 3.0.2.

## Results

We observed no difference in in patients characteristis and baseline spirometry results between the two ISB approaches (Table 1).

**Table 1:** Patients’ characteristics by puncture approach at baseline

Patient characteristic	Anterior puncture approach	Posterior puncture approach
	*N*_total_ = 42	*N*_total_ = 42
Nfemales (%)	**12 (29 %)**	**13 (31 %)**
Median age [years]	51	50
(Q1, Q3)	(43, 56)	(41, 56)
Median Body Mass Index [kg/m^2^]	26	28
(Q1, Q3)	(25, 29)	(25, 31)
N_surgical procedure_ (%)		
Arthroskopic labrum refixation	23 (55)	22 (52)
Open labrum refixation	13 (31)	13 (31)
Other	6 (14)	7 (17)
N_diagnosis_ (%)		
Tendinosis calcacera	14 (33)	18 (43)
Lesion of the rotator cuff	16 (38)	13 (31)
Bankart lesion	10 (24)	6 (14)
Other	2 (5)	5 (12)
Median spirometry variables at baseline (Q1, Q3)		
VC [l]	5.09 (3.98, 5.58)	4.46 (3.90, 5.37)
FEV1 [l/s]	3.8 (3.34, 4.33)	3.57 (3.01, 4.11)
Sniff PmaxPeak [kPa]	6.24 (4.84, 7.67)	6.25 (5.09, 8.63)

At the time of the second spirometry, all patients except one showed a complete motor block of the upper extremity. One patient from the posterior approach group needed a general anesthetic due to an insufficient nerve block. In all other patients the operation could be performed under regional anesthesia and sedation resulting in a total block success rate of 98.8 % (95 % CI: 96.5–100.0 %).

**Fig. 3:**
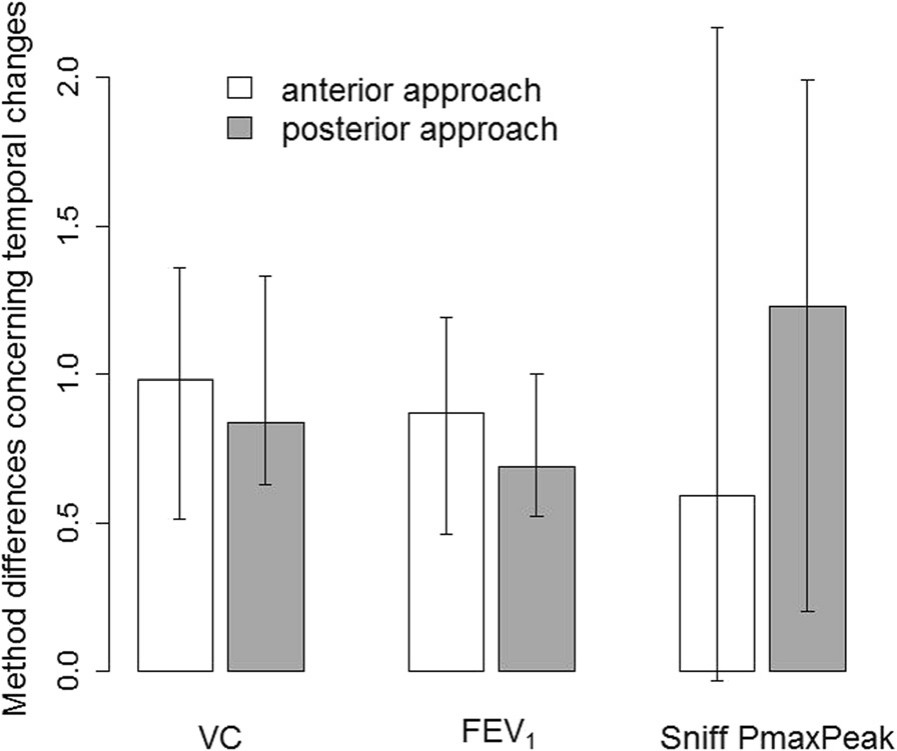
Median temporal differences between anterior and posterior puncture approach for spirometry outcomes with 95 % confidence intervals

Compared to the initial value collected from baseline spirometry, the second spirometry showed a significant decrease of VC, FEV_1_ and Sniff P_maxPeak_ in both approaches indicating phrenic nerve blocks (Table 2). Neither a difference in the spirometric parameter development from T_0_ to T_30_ nor a difference in the incidence of hemidiaphragmatic paresis could be observed between the anterior and the posterior approach groups (Table 3, Fig. 3). The sensitivity analysis revealed no difference between the puncture approaches at the two time points (Table 3)

**Table 2:** Results of the spirometry outcomes for the anterior and posterior puncture approach comparing baseline (T_0_) and 30 min after the interscalene block (T_30_)

		Anterior puncture approach			Posterior puncture approach		
Spirometry variable	Time	Median (Q1, Q3)	Median difference_T0-T30_ (95 % CI)	*p*-value (two-sided)	Median (Q1, Q3)	Median difference _T0-T30_ (95 % CI)	*p*-value (two-sided)
VC [l]	T_0_	5.09 (3.98, 5.58)	0.95 (0.79, 1.11)	<0.001	4.46 (3.90, 5.37)	0.96 (0.81, 1.14)	<0.001
	T_30_	3.98 (3.31, 4.67)			3.46 (2.90, 4.34)		
FEV_1_ [l/s]	T_0_	3.8 (3.34, 4.33)	0.75 (0.63, 0.87)	<0.001	3.57 (3.01, 4.11)	0.83 (0.70, 0.99)	<0.001
	T_30_	3.05 (2.41, 3.44)			2.58 (2.05, 3.22)		
Sniff PmaxPeak [kPa]	T_0_	6.24 (4.84, 7.67)	1.10 (0.71, 1.56)	<0.001	6.25 (5.09, 8.63)	1.07 (0.46, 1.74)	<0.001
	T_30_	5.13 (4.21, 7.22)			5.70 (4.54, 6.83)

**Table 3:** Results of the spirometry outcomes at baseline (T_0_) and 30 min after the interscalene block (T_30_) comparing the anterior and the posterior puncture approaches

Spirometry variable	Puncture approach	T_0_			T_30_		
		Median (Q1, Q3)	Median difference_anterior-posterior_ (95 % CI)	*p*-value (two-sided)	Median (Q1, Q3)	Median difference_anterior-posterior_ (95 % CI)	*p*-value (two-sided)
VC [l]	anterior	5.09 (3.98, 5.58)	0.38 (−0.15, 0.88)	0.167	3.98 (3.31, 4.67)	0.40 (−0.08, 0.88)	0.088
	posterior	4.46 (3.90, 5.37)			3.46 (2.90, 4.34)		
FEV1 [l/s]	anterior	3.80 (3.34, 4.33)	0.25 (−0.16, 0.63)	0.174	3.05 (2.41, 3.44)	0.34 (−0.09, 0.72)	0.103
	posterior	3.57 (3.01, 4.11)			2.58 (2.05, 3.22)		
Sniff PmaxPeak [kPa]	anterior	6.24 (4.84, 7.67)	−0.24 (−1.24, 0.83)	0.655	5.13 (4.21, 7.22)	−0.23 (−1.04, 0.56)	0.582
	posterior	6.25 (5.09, 8.63)			5.70 (4.54, 6.83)

When skin suturing was started, the sedation was terminated. Upon arrival in the recovery room, 80 patients (95.2 %; 95 % CI: 90.7–99.8 %) already had an Aldrete score of ten; three patients (3.6 %; 95 % CI: 0.0–7.5 %) had a score of nine, and one patient (1.2 %; 95 % CI: 0.0–3.5 %) had a score of eight. A subjective dyspnea did not occur in any of these patients.

## Discussion

As assessed by spirometry there was no difference with regards to phrenic nerve block between the anterior or posterior interscalene approach using a total of 15 ml ropivacaine 1 % for each ultrasound guided block. Despite the fact that our sample size calculation was based on a power of 80 % we do not believe we have missed any relevant effect of the two different approaches in this setting because we decided to include additional patients to adress a potential drop out-rate and to give the trial more power. More details were given in the method section.

Another explanation for the absence of significance is that the volume used seems too large not to spread towards the phrenic nerve. Subsequently, the anterior or posterior site of injection cannot reduce the incidence of unintentional hemidiaphragmatic paresis in this setting.

In order to avoid phrenic nerve blocks, a reduction of the local anesthetic volume comes into consideration. Both in medical journals and in clinical practice you will find data regarding injection volumes between 20 and 50 ml [12, 13], which are higher than the 15 ml used in this study. Meanwhile, several studies have shown that the minimum effective anesthetic volume (MEAV) for a sufficient interscalene plexus block is far below the current clinical volumes. McNaught et al. reported an MEAV of 0.9 ml [14]. However, the operations were performed under general anesthesia, and the outcome of an effective block was postoperative analgesia 30 min after the end of surgery. Falcao et al. reported a similar MEAV of 0.95 ml. After having performed a preoperative sensory and motor evaluation on the block, a general anesthesia was also performed [15]. The results of a dose-finding study by Gautier et al. appear to have pioneered the interscalene plexus block and the subsequent operation using 5 ml ropivacaine 0.75 % (ca. 1.7 ml per root) without general anesthesia [16]. The minimum effective volumes for ultrasound guided interscalene blocks associated with a general anesthesia, which were reported by McNaught and Falcao [14, 15], reveal that the volume of 15 ml used in our study was far beyond the dose required. These results had not yet been published at the time of planning for the present study (2011).

As already shown by Gautier et al. [16], the injection volume can certainly be considerably reduced in an interscalene block with sedation and without general anesthesia for shoulder operations. It would be useful to keep a concentration of 1 % because probably the volume, not the administered concentration of the local anesthetic, causes problems. In 2009 Renes at al. described that the usage of an ultrasound guided supraclavicular brachial plexus block results in an up to 100 % avoidance of an undesirable hemidiaphragmatic paresis (*n* = 30) [6]. However, the block was exclusively used in elbow, forearm, wrist, and hand surgery. Case studies by Erickson and Chaudhuri et al. report that also here hemidiaphragmatic paresis cannot always be avoided [17, 18]. Erickson published a surgical study, however, without any detailed data concerning the anesthesiologic technique or the injected volume. The second case study reports an injection of 20 ml [17].

In our study, an injection site located more distally towards the supraclavicular region could also be considered for shoulder operations in ultrasound guided brachial plexus block and sedation, in addition to the previously discussed reduction of the injection volume. In a “deep interscalene” or rather “supra-supraclavicular” position, the same parts of the brachial plexus are accessible as in an interscalene position. Particularly the suprascapular nerve and the dorsal scapular nerve are still accessible here, whereas the phrenic nerve takes a course in much larger distance. The route relating to C_5_ and the anterior scalene muscle can be displayed well sonographically [19]. Therefore it is postulated that the paresis rate can be effectively reduced by the up to now unusual injection site and also by the reduction of the injection volume. Verelst et al. suggested a similar strategy in 2013 [20]. This approach gains relevance from a study by Kaufman et al. [21], who described a case series of direct phrenic nerve injuries following interscalene blocks [21, 22].

In our study, the block success rate of 98.8 % is very high and is in the same range as in data published by Bishop et al. in 2005 where it is 97 % [23]. There, neither the injection site nor the approach plays a role. As Lang et al. demonstrated in 2012, there is no discrepancy in the effect of the block whether the nerve roots are only partially or entirely flushed with the local anesthetic [24]. Spence et al. [25] reached a similar conclusion when they showed that an exact injection site of the local anesthetic does not make any difference regarding the effect of the block.

Despite a significant deterioration of the pulmonary function, the operation could be performed on 98.8 % of our patients without general anesthesia. Likewise the monitoring in the recovery room and the postoperative course within the first 24 h after the block proved uneventful from a clinical point of view. This supports the clinical experience that although a hemidiaphragmatic paresis frequently occurs, it is not clinically relevant in the majority of cases in patients with normal pulmonary mechanics. Against the background of the study results, it can be assumed that ≤ ASA II patients without preexisting pulmonary disease are not clinically impaired by an interscalene block-induced phrenic nerve block with subsequent hemidiaphragmatic paresis. Despite the paresis, they can be moved directly from the operating room to the general patient care unit, provided that they have an Aldrete score of ten upon leaving the operating room.

However, our study has several limitations including a relatively small sample size and perhaps too large volume of local anesthetic. Furthermore, the spirometric outcomes are dependent on both the patient’s cooperation and the examiner’s instructions. In addition, a learning effect in patients cannot be excluded between the first and the second spirometry. This might have influenced the accuracy of diagnosing hemidiaphragmatic paresis.

From a sensitivity and specificity viewpoint, magnetic stimulation on the phrenic nerve is considered the gold standard for the diagnosis of diaphragmatic paresis. However, this procedure can only be carried out under study conditions in a few clinical centers because it is a technical and highly invasive specialized procedure, due to the placement of an esophageal pressure tube. From an ethical point of view, radiation exposure induced by a radiological examination of the diaphragm was not considered reasonable for the patients in this study.

## Conclusion

We found the ultrasound guided anterior approach of the interscalene block to be equally effective to the posterior approach when an injection volume of 15 ml ropivacaine 1 % was used. There was no difference in the incidence of hemidiaphragmatic paresis. Clinical breathing effects of phrenic nerve blocks are, however, usually well compensated, and subjective dyspnea did not occur in our patients. Future studies could be aimed at a further reduction of the injected local anesthetic volume in order to reduce the incidences of hemidiaphragmatic paresis.

## Abbreviations

ASA, American Society of Anesthesiologist physical status classification system; FEV_1_, forced expiratory volume per second; GOLD, Global initiative for chronic Obstructive Lung Disease; ISB, interscalene brachial plexus block; Sniff, maximum nasal inspiratory breathing maneuver; VC, vital capacity
